# Testis Development and Differentiation in Amphibians

**DOI:** 10.3390/genes12040578

**Published:** 2021-04-16

**Authors:** Álvaro S. Roco, Adrián Ruiz-García, Mónica Bullejos

**Affiliations:** Departamento de Biología Experimental, Facultad de Ciencias Experimentales, Campus Las Lagunillas S/N, Universidad de Jaén, 23071 Jaén, Spain; asroco@ujaen.es (Á.S.R.); arg00027@red.ujaen.es (A.R.-G.)

**Keywords:** amphibian, sex determination, gonadal differentiation, testis, sex reversal

## Abstract

Sex is determined genetically in amphibians; however, little is known about the sex chromosomes, testis-determining genes, and the genes involved in testis differentiation in this class. Certain inherent characteristics of the species of this group, like the homomorphic sex chromosomes, the high diversity of the sex-determining mechanisms, or the existence of polyploids, may hinder the design of experiments when studying how the gonads can differentiate. Even so, other features, like their external development or the possibility of inducing sex reversal by external treatments, can be helpful. This review summarizes the current knowledge on amphibian sex determination, gonadal development, and testis differentiation. The analysis of this information, compared with the information available for other vertebrate groups, allows us to identify the evolutionarily conserved and divergent pathways involved in testis differentiation. Overall, the data confirm the previous observations in other vertebrates—the morphology of the adult testis is similar across different groups; however, the male-determining signal and the genetic networks involved in testis differentiation are not evolutionarily conserved.

## 1. Introduction

The class Amphibia includes 8301 species in three orders, with distinct representation and anatomical features: 88% Anura (frogs and toads), 9% Caudata (salamanders and tritons), and 3% Gymnophiona (caecilians, or limbless amphibians) [1]. Despite their worldwide decline, new species are discovered every year (60% increase in the number of species since 1985) [1].

Amphibians constitute an interesting group in which reproductive successful hybrids can be produced, and polyploidy (natural or artificial) is well tolerated. Natural polyploids have been described in 15 anuran and four urodelan families, whereas (to the best of our knowledge) no polyploid species have been identified in caecilians (for a thorough review on amphibian polyploid species, see [2]). True parthenogenesis does not exist in this class, but there are examples of unisexual and bisexual species reproducing by hybridogenesis (*Pelophylax* kl. *esculentus* [3]), kleptogenesis (unisexual salamanders of the genus *Ambystoma* [4]), gynogenesis (induced [5,6], not demonstrated in nature), and pre-equalizing hybrid meiosis (*Bufo pseudoraddei baturae* [7]). Amphibians show developmental plasticity in response to environmental conditions [8] and a great variety of reproductive modes [9]. Their development includes an embryonic period and a larval phase that ends in metamorphosis. The majority of amphibian species are oviparous, with the eggs developing in a wide range of environments (e.g., water, foam, plants, or even in the back or the stomach of the adults). Viviparous and ovoviviparous species exist, mainly in urodelan and caecilians, with the eggs developing in the oviducts. These characteristics can be beneficial for research, but can also impair the analysis of gonadal development in this clade.

In this review we will cover the process of testis development in amphibians, from the appearance of the gonad in the body cavity to testis differentiation at metamorphosis. Testis differentiation in juvenile and adults in the class Amphibia is thoroughly covered in [10]. Before starting with gonadal development, we have included one section about the sex chromosomes and sex-determining genes in this group, highlighting those characteristics that hinder the analysis of gonadal development and differentiation.

## 2. Amphibian Sex Chromosomes and Sex Determination

### 2.1. Sex Chromosomes

In general, the males are the heterogametic sex in mammals (XX/XY), whereas, in birds, it is the females (ZZ/ZW). The situation in amphibians is not as simple. Both male (XX/XY) and female (ZZ/ZW) heterogamety can be found in this class, and transitions between the same or different sex chromosome systems are identified even in closely related species (e.g., the family Ranidae [11], the genus *Xenopus* [12,13], and the genus *Bufo* [14]). Other combinations are also possible, like systems with multiple sex chromosomes [15,16,17], with the extreme example of the six pairs of sex chromosomes described in *Leptodactylus pentadactylus* [18], or the 00/W0 female heterogamety identified in *Leiopelma hochstetteri* [19]. To further complicate the situation, most amphibian species have homomorphic sex chromosomes (for a review, see [20,21,22]). According to The Tree of Sex Consortium [23], the sex chromosomes have been analyzed in about 2% of amphibian species (114 Anura, 58 Caudata and 1 Gymnophiona), identifying sex chromosome heteromorphism in 38% of the analyzed species (17% in Anura, 45% in Caudata and in the only Gymnophiona analyzed) [23].

The identification of the sex chromosome system operating in one species is not straightforward if the sex chromosomes are homomorphic. In that case, this information can be obtained by analyzing the sex ratio in the offspring produced by gynogenesis, or from crosses that involve sex-reversed individuals (e.g., *Pleurodeles waltl* [24] and *Xenopus laevis* [25,26]). These results are not always straightforward, and bizarre sex ratios may reveal non common sex-determining mechanisms. This is the case of the frog *Fejervarya kawamurai*, where a multifactorial complementary sex determination has been proposed [27]. With the advent of genome sequencing strategies, sex-linked markers (and sex heterogamety) have been identified in a growing number of amphibian species. These markers reveal a lack of conservation in the sex chromosomes in this group and show that specific chromosomes have evolved independently as sex chromosomes in different lineages. These “preferred” sex chromosomes harbour sex-related genes like *dmrt1*, *sox3*, *ar*, and *foxl2* [11,28,29,30,31].

Geographical variations in sex chromosomes have been described in single species (e.g., *Rana temporaria* [32,33], *Glandirana rugosa* [34,35], and *Xenopus tropicalis* [36]) and should be taken into account when analyzing gonadal development, since different populations can have different sex-determining genes. The most extreme example occurs in *G. rugosa*, in which six geographic variants with both XX/XY and ZZ/ZW sex chromosomes evolved after two independent chromosomal inversions followed by hybridization events [37,38,39,40,41,42]. Another example of intraspecies variation occurs in the amphibian model *X. tropicalis*, a species with three sex chromosomes (Y > W > Z) coexisting in laboratory strains [36] and in natural populations [43], which likely originated after the emergence of a Y chromosome from an ancestral Z chromosome [43].

### 2.2. Sex Determination

Amphibian sex determination is presumed to be controlled genetically [34,44,45,46,47,48]. However, it is also evident that environmental cues, such as temperature or steroid hormones, can override genetic sex determination, producing sex reversal [49,50,51]. Sexual steroids, estrogens, and androgens can easily induce sex reversal (for a review, see [45,49,50]). On the other hand, though thermal effects on sex determination have been considered anecdotal under natural conditions [45], high and low temperatures can induce sex reversal in anuran and urodelan species (e.g., *Rana chensinensis* [52], *Quasipaa spinose* [53], *Fejervarya limnocharis* [54], *Xenopus* polyploid hybrids [55], *Hynobius retardatus* [56], *P. waltl* [24], *Pleurodeles poireti* [57], *Triturus cristatus* [58], and *Triturus carnifex* [50]) (for a recent review, see [59]). The implication of environmental factors in sex reversal in natural populations of amphibians has been underscored, likely due to the lack of knowledge regarding the sex chromosomes or the sex-determining genes in most amphibian species. Indeed, the increasing number of molecular sex-linked markers suggests that sex reversal may be more common than suspected in amphibians (sex-reversed individuals have been described in natural populations of the species *Rana clamitans* [60] and *R. temporaria* [61]).

The genes involved in sex determination in amphibians are largely unknown, partly due to the difficulty in identifying the sex chromosomes in most species. In addition, homomorphic sex chromosomes facilitate the rapid turnover of sex-determining genes [62]. This could explain why so many different sex-determining genes have been identified in fish (*dmy*, *Sdy*, *Gsdfy*, *Sox3y*, *amhy*, *amhr2y*, *Dmrt1*, and *gsdf6y* (for a review, see [63,64])), a group also characterized by a high frequency of homomorphic sex chromosomes, and foreshadows a similar situation in amphibians [65,66].

The only sex-determining gene known in this class, the *dm-w* gene, is also the only W-linked sex-determining gene identified in vertebrates [67]. It was first described in *X. laevis*, an allotetraploid species with homomorphic ZZ/ZW sex chromosomes [68,69], which originated after the hybridization of two ancestral species (genomes L and S) with 2n = 18 chromosomes each (2n = 4x = 36, LLSS) [70]. The *dm-w* gene evolved from a partial duplication of *dmrt1*.S (on chromosome 1S), occurring after allotetraploidization [71,72]. *dm-w* shows specific expression in ZW bipotential gonads, and its role in *X. laevis* sex determination was confirmed by functional analysis. The overexpression of *dm-w* can induce female differentiation in ZZ tadpoles, whereas *dm-w* knockdown can cause female-to-male sex reversal in ZW larvae [67,73]. The precise mechanism of action of *dm-w* is still unknown. It has been proposed that Dm-w functions as a dominant-negative form of Dmrt1 (lacking the transactivation domain), antagonizing the testis formation promoted by Dmrt1 and resulting in a high expression of *cyp19a1* and *foxl2* [74].

*dm-w* is present in other species of the genus *Xenopus* (though not in *X. tropicalis*) [13,72], but its involvement in sex determination in these species has not yet been experimentally confirmed. In those species in which *dm-w* is not completely female-specific, this gene may be a weak determining gene, or not necessary to determine female development [13]. In addition, species with a female-specific *dm-w* gene may have differences in their sex-determining pathways. This is likely the case in *X. laevis* and *X. gilli*, two species with the same chromosome number (2n = 4x = 36) that can hybridize in nature [75,76,77]. Both species have a ZZ/ZW sex chromosome system and a female-specific *dm-w* gene [13,72]. There is no information regarding the sex-determining ability of *dm-w* in *X. gilli*, but the sex ratios of polyploid hybrids show that the sex-determining pathways may not be equivalent in both species (the frequency of males with W^L^ZZ genotypes depends on the species that provided the Z chromosomes) [55,78].

No other sex-determining gene has been identified in amphibians. Several lines of evidence have proposed a critical role of the androgen receptor (*ar*) as a male-determining gene in *G. rugosa* [79,80], although functional experiments do not fully corroborate this role [30,81]. Thus, it remains unclear which gene is required for sex determination in this species. The existence of different sex-determining loci in different populations should be also taken into account, since they can provide an explanation for the 1:1 sex ratios observed among the XZ offspring of crosses between XX females and ZZ males from different populations [82].

Autosomal factors may also be involved in sex determination in amphibians (i.e., *F. kawamurai*) [27]. This could be the case for the Japanese frog *Buergeria buergeri*, a species with heteromorphic ZZ/ZW sex chromosomes [83] where triploid ZZW individuals (n = 80) can be either male (53.8%) or female (46.2%) [84]. An alternative explanation could be that sexual differentiation proceeds randomly in either the male or female direction if one W and two Z chromosomes cannot tilt the balance toward the male or the female pathway in ZZW individuals [85].

## 3. Gonadal Development and Differentiation in Amphibians

### 3.1. General Considerations

The general model of gonad development appears to be universal for all vertebrates. However, variations in particular vertebrate groups, or even in species, are evident. In amphibians, gonadal formation takes place during the larval stages; however, not all amphibian species have differentiated gonads at metamorphosis, as the development of testes and ovaries progresses at different paces in different species [86]. The rates of gonadal differentiation vary considerably among species and sexes. Depending on the stage of differentiation at metamorphosis, three rates can be established in the ovaries: the basic rate (ovarian cavity appears at the end of metamorphosis), delayed rate (the first diplotenic oocytes arise after metamorphosis), and accelerated rate (pre-vitellogenic oocytes appear before metamorphosis) [87]. Correspondingly, three rates can also be considered during testicular development based on the timing of the differentiation of seminiferous tubules: the basic rate (during metamorphosis), delayed rate (after metamorphosis), and accelerated rate (before metamorphosis) [88].

Undifferentiated gonads in most amphibian species differentiate directly into ovaries or testes. This pattern of gonadal differentiation has been called differentiated or undifferentiated, depending, respectively, on the simultaneous or delayed differentiation of the testes compared with the ovaries [89]. In certain species or populations (sexually undifferentiated races, according to [90]), it has been reported that undifferentiated gonads of both sexes undergo a secondary undifferentiated condition of ovarian type before gonadal differentiation. In this pattern of gonadal differentiation (called semi-differentiated by Gramapurohit [89]), testes will develop in males after oocyte degeneration. A semi-differentiated pattern has been described in several anuran species (e.g., *Rana sylvatica*, *Rana dalmatina*, *Rana latastei*, *Rana curtipes*, and *Rachophorus arboreus* [91] and the references therein). It has been argued that undifferentiated testes in amphibians do not go through an ovary-like transition phase [87,92]. However, the existence of this transition phase cannot be completely ruled out, considering the information available on undifferentiated races (*sensu* Witschi) [93,94]. In addition, a similar condition (called “juvenile hermaphroditism”) was described in zebrafish (*Danio rerio* [95]) and black tetra (*Gymnocorymbus ternetzi* [96]). In these species, during gonadal development, most individuals develop undifferentiated ovary-like gonads [97,98]. The transformation of these gonads into testes is characterized, as in amphibians, by the apoptosis of early diplotenic oocytes [97]. The apoptosis of oocytes could be triggered by a decrease in aromatase, as lower levels of aromatase were detected in early diplotenic oocytes from undifferentiated ovary-like gonads changing to testes [97]. This possibility is supported by the results obtained after treatment with fadrozole, as this aromatase inhibitor induces oocyte apoptosis and female-to-male sex reversal [97,99]. In this regard, it would be of interest to analyze the expression pattern of the genes involved in gonadal differentiation in species with an alleged semi-differentiated pattern. In this way, it would be possible to check if the initiation of the female pathway takes place in these species before the onset of the male pathway.

### 3.2. Sexually Undifferentiated Gonad

#### 3.2.1. Germ Cell Specification

Germ cells constitute an essential part of the gonad, although they originate outside it. Two modes of germ cell specification exist in animal embryos [100], and both can be found in amphibians [101]. In anurans, primordial germ cells (PGCs) have an endodermal origin and are specified through preformation/inheritance, as a consequence of the localization in the egg of maternally inherited germ cell determinants (germ plasm) [102]. In urodeles, the PGCs are of mesodermal origin and the segregation of the germ cell lineage among somatic cells occurs as it does in mammals, through cell–cell interactions (inducing signals secreted by embryonic tissues) [103,104]. In Gymnophiona, the PGCs are located at a young stage in the endoderm [105], although not much is known about their specification.

Inductive germ cell determination is likely an ancestral mode of germ cell specification, whereas inherited specification is a derived mechanism that evolved through convergence. Thus, the acquisition of the germ plasm must have evolved independently in several lineages of vertebrate embryos, including anurans. The evolutionary cause for the convergence of the germ plasm is still under debate [100,106].

#### 3.2.2. Initial Gonadal Formation

In amphibians, the presumptive gonads originate from two longitudinal thickenings of the celomic epithelium, localized on the ventral part of the mesonephros along both sides of the gut mesentery (reviewed in [45,49,92,105]). As in other vertebrate species, primordial germ cells (PGC) of extragonadal origin invade the gonadal anlagen, forming undifferentiated gonads composed of germ and somatic cells. In some amphibian species, genital ridges only become evident when the PGCs arrive at the presumptive gonad (e.g., *Bombina orientalis* [107], *X. laevis* [108], and *Bombina variegata* [109]). However, genital ridges can develop independently of germ cells, as sterile gonads can differentiate in the absence of these cells (e.g., *X. laevis* [110,111], *Xenopus* hybrids [112], *P. waltl* [113]).

Initially, the gonads are bipotential and no histological differences between sexes can be identified [49]. The bipotential gonads in amphibians differ from those of birds and reptiles by the absence of primitive sex cords (for a review, see [114]). However, as in birds and reptiles, they are compartmentalized into two domains: the cortex, formed by somatic cells and gonia, and the medulla, formed only by somatic cells (e.g., *Bufo bufo* [91], *R. nigromaculata* [115]). The presence of these two domains is common in amphibians, although the onset of their separation is species-specific (e.g., *B. orientalis* [107], *B. variegata* [109], *Ambystoma mexicanum* [116], and caecilians [105], reviewed in [92]), and independent of the presence of germ cells [110].

### 3.3. Sexual Differentiation of the Testis

#### 3.3.1. Morphological Changes

The fate of the bipotential gonad is established during sex determination. Despite the lack of conservation in the sex-determining gene in this group, one of the first morphological signs of sexual differentiation, the change in the localization of the germ cells in males, is quite conserved. In the differentiating testes, the germ cells migrate from the cortex into the medulla, where they interact with differentiating Sertoli cells (precursor Sertoli cells) and peritubular myoid cells, forming cysts. Thus, developing testes are easily distinguishable due to the lack of a clear compartmentalization into the cortex and medulla: the sterile cortex becomes a thin layer that surrounds the gonad (the tunica albuginea), whereas the germ cells (spermatogonia) are distributed through the entire gonad (e.g., *B. bufo* [91], *Rana nigromaculata* [115], *X. laevis* [117], *R. temporaria*, *Hyla arborea* and *Pelophylax lessonae* [118], and *P. waltl* [104]). In the differentiating ovaries, oogonia remain in the cortex, where they proliferate and form follicles when surrounded by somatic cells, whereas a medulla without germ cells forms in the center of the gonad. Soon after the onset of differentiation in the ovary, the medulla is completely modified, with medullary cells losing cell junctions and resulting in the formation of ovarian cavities, which are absent in the testes (e.g., *B. bufo* [91], *R. nigromaculata* [115], *X. laevis* [117], and *P. waltl* [104]); for a review, see [92].

Depending on the species, the germ cells and their migration from the cortex to the medulla are not required for testis cord formation or for testis differentiation (e.g., *X. laevis* [110], *Xenopus* hybrids [112], *P. waltl* [113]). However, germ cells may play important roles in the development of the ovaries and the maintenance of the ovarian structures. In fact, ZW tadpoles from *X. laevis* develop male gonads when their germ cell number is reduced after knocking down the germ cell-specific form of *dmrt1*.L [119].

The origin of the somatic cells of the gonads is controversial in amphibians [45,49]. Ultrastructural analysis has suggested that somatic cells in both the seminiferous cords and follicles are derived from the celomic epithelium [91,93,109,115,120,121]. The origin of the medulla is less evident. It has been proposed that the mesonephric blastema [104,122] or the interrenal gland [123] contribute cells to the medulla. However, most studies state that, in anurans, the medulla is derived from the celomic epithelium (e.g., *B. bufo* [91], *Rhacophorus arboreus* [93], *B. variegata* [109], *R. nigromaculata* [115], *Rana pipiens* and *X. laevis* [121]). The rete testis is formed from medullary cords near the hilus. Thus, it is not unusual that species that lack a sterile medulla during testicular differentiation also lack the rete testis (e.g., *B. variegata* [109]). Finally, mesenchymal cells and blood vessels migrate from the mesonephros into the gonads [117,121]. They are located between the cortex and the medulla, with basal lamina forming at the interface of the celomic epithelium-derived cells and the stromal cells. In this way, different gonadal structures are achieved in both sexes ([117] and the references therein).

#### 3.3.2. Cellular Mechanisms Involved in Testis Differentiation

The development of the testis in vertebrates occurs through a series of common cellular mechanisms, although differences between groups or species are possible. The proliferation of Sertoli cell precursors is one of the first morphological differences observed between the sexes in mice [124], chickens [125], and turtles [126], indicating that it may be a conserved mechanism in vertebrate testis organogenesis [127]. Proliferation has been studied in a wide variety of anuran (*X. laevis*, [117], *R. nigromaculata* [120], *G. rugosa* [128]) and urodelan (*A. mexicanum* [116], *P. waltl* [129]) species. However, its role in testis differentiation in this class is not evident. The proliferative activity in undifferentiated amphibian gonads is more marked in the proximal regions, including the gonadal mesentery and medulla; however, no differences were observed between both sexes before differentiation began. In differentiated gonads, sex-specific cell proliferation patterns can be observed, with the testes showing somatic proliferation throughout the gonads, whereas proliferation occurs in proximal locations in developing ovaries [117,128].

Another cellular mechanism involved in testis differentiation in certain species is the migration of mesonephric cells into the gonads. Mesonephric cell migration is required for testis cord formation in mice [130,131]. However, cells migrating from the mesonephros do not appear to be necessary for testis cord formation in the sea turtle (*Lepidochelys olivacea*), as bipotential gonads separated from the mesonephros showed testis cord development [132]. The existence of primitive sex cords in the bipotential gonads in turtles, but not in mice, could explain the requirement of mesonephric cell migration for testis cord formation in mice, but not in turtles [126]. In fact, although male-specific mesonephric cell migration is conserved in both mice and chickens, its inhibition has no effect on the testis cord organization in chickens, a species that also retains primitive sex cords in undifferentiated gonads [133,134]. In amphibians, migration from the mesonephros occurs in both sexes, but only after gonadal differentiation [117,122,128]. Thus, differences in mesonephric migration in vertebrates (regarding (1) its onset, (2) its requirement for testis cord formation, and (3) the sex of the gonads where mesonephric cells migrate) may be the consequence of different requirements to form or maintain testis cords in developing gonads. This, in turn, could depend on (1) the presence or absence of primitive cords in bipotential gonads and (2) the presence or absence of compartmentation of undifferentiated gonads in the cortex and medulla. Investigating the requirement of mesonephric cell migration for the formation of testis cords in amphibian male gonads will provide information about the evolutionary dynamics of vertebrate testis differentiation.

#### 3.3.3. Germ Cell Differentiation: Spermatogenesis

The sex of the germ cells depends on the sex of the developing gonads. In amphibians, as in all vertebrate groups, the onset of meiosis depends on the developmental pathway of the gonad. Germ cells enter meiosis in the larval ovary; however, meiosis is generally delayed until after metamorphosis in males [87,88,135]. Exceptions exist, and species have been described that produce sperm at the end of metamorphosis, including *Pseudis paradox* and *Pseudis minuta* [136,137].

The somatic cells, specifically Sertoli cells, are responsible for providing the correct environment for germ cell proliferation, meiosis, and sperm differentiation (spermatogenesis) [138,139] (for detailed reviews, see [140,141,142]). In vertebrates, two major types of spermatogenesis can be identified based on the structure of the basic unit where it takes place. In amniotes (mammals, birds, and reptiles), spermatogenesis occurs in the seminiferous tubules (non-cystic spermatogenesis), whereas in anamniotes (amphibians and fish) spermatogenesis takes place in cysts, a structure formed when Sertoli cells engulf a single spermatogonia stem cell (for a discussion about the cyst concept, see [143] and references therein).

Spermatogenesis in amphibians has been divided into prespermatogenesis and active spermatogenesis [144], in the same way it has been described for humans [145]. During prespermatogenesis the gonocytes (also called prespermatogonia or prospermatogonia [146]) proliferate in the developing testes of tadpoles, surrounded by pre-Sertoli cells. On the other hand, during active spermatogenesis, spermatogonial stem cells inside the cyst either proliferate (self-renewing the spermatogonial pool) or produce secondary spermatogonia that enter meiosis (primary and secondary spermatocytes), yielding round spermatids and then sperm [10,144]. According to this division, primary spermatogonia can be prespermatogonia (in larval testes, equivalent to gonocytes in mammals) or spermatogonial stem cells (in juvenile and adult testes), two cell types showing distinct ultrastructural morphology [144].

Information about amphibian spermatogenesis mostly cover active spermatogenesis (e.g., *Pelophylax bedriagae* [147], *P.* kl. *esculentus* [148], *Salamandra salamandra* [149]) or spermiogenesis (*Odontophrynus cultripes* [150], and *Ambystoma dumerilii* [151]), whereas prespermatogenesis has been only studied in *P. lessonae* and *Pelophylax ridibundus* [144]. For a detailed description of spermatogenesis in the three amphibian orders, see [10,152].

Sertoli cells differentiate and proliferate as the cyst grows and matures. The number of Sertoli cells forming a given cyst grows with the mitotic division of the germ cells. Once germ cells enter meiosis, proliferation ceases, coinciding with the formation of tight junctions and desmosomes between Sertoli cells (the testis barrier is formed). Once the spermiogenesis has finished, the cysts open to release spermatozoa during spermiation. In this process Sertoli cells degenerate in urodelan amphibians [140]. In anurans, the basal parts of the Sertoli cells remain after spermiation, later regenerating the Sertoli cells [153]. The transient germinal epithelium characteristic of cystic spermatogenesis requires the turnover of Sertoli cells. Sertoli cells can divide when they are in contact with mitotically active spermatogonia stem cells. In this way, new cysts are formed periodically. It has been hypothesized that a population of Sertoli stem cells must exist in amphibians, as it exists in the transition zone (where seminiferous tubules connect to the rete testis) in mammals [154], but evidence is still lacking [155]. Most data on the control of Sertoli cell proliferation come from observations in fish (e.g., *Oreochromis niloticus* [156,157], *Clarias gariepinus* [158], *D. rerio* [159]; for a review, see [141]). In this group two regulatory mechanisms of Sertoli cell proliferation have been described: (1) to generate new cysts (stimulated by thyroid hormone/FSH and estrogen through high levels of Igf3 and Pdecgf), and (2) during the development of existing cysts (activated by FSF and progestins through Igf3 and androgens produced by Leydig cells) [155]. The existence of equivalent regulatory pathways in amphibians must be corroborated experimentally.

The spermatogonial stem cells in the cyst proliferate by mitoses. Due to incomplete cytokinesis, cytoplasmic bridges keep germ cells connected (a characteristic widely conserved across animals). As a consequence, all the germ cells enclosed in one cyst are at the same developmental stage, although examples of asynchrony in the same cyst have been described (e.g., *P. lessonae* and *P. ridibundus* [144]). It has been proposed that the asynchrony could be due to the breakage of cytoplasmic bridges (e.g., due to cell death) or to the inclusion of two germ cells in the same cyst [144].

Spermatogenic efficiency (and the Sertoli supporting capacity) has been reduced over the course of vertebrate evolution [155]. The change from cystic to non-cystic spermatogenesis involves an increase in regulatory complexity in Sertoli cells (these cells provide a supporting environment for germ cells at different developmental stages at the same time, e.g., cells located in basal, medial, and adluminal regions of the seminiferous tubules) and a reduction in the Sertoli supporting capacity. On the other hand, cystic spermatogenesis supports the development of more spermatogonia, producing a higher amount of spermatozoa. In fact, the supporting capacity of Sertoli cells is 10 times higher in anamniotes than in mammals [155]. In this sense, greater attention should be paid to the effect of environmental contaminants on the supporting capacity of Sertoli cells in amphibians and on the fertility of these species.

## 4. Testis Plasticity

Most caudata are atypical regarding testis differentiation. In Salamandridae and Plethodontidae, males possess multi-lobulated testes that continue to differentiate during adult life [160]. In the differentiated testis of *P. waltl,* two regions can be identified: (1) the undifferentiated anterior region, a source of germ cells for the differentiated lobe, although it can also form a functional testis if the differentiated lobe is removed [160]; and (2) the first lobe, located in the posterior region of the developing gonad at the end of metamorphosis. New lobes can form periodically after the extrusion of spermatozoa and regression of the empty part of the testis. This triggers differentiation of adjacent Leydig cells and the formation of the glandular region in the last lobe. Quiescent germ cells present in the glandular region become spermatogonia and generate another testis lobe in the caudal position [160].

Another particularity of testis development in certain amphibian species (the Bufonidae family) is Bidder’s organ [161,162,163], an ovary-like gonadal structure located at the most anterior part of male and female developing gonads (for a description of the morphological diversity of Bidder’s organ and adult gonads in bufonids, see [162]). The development of Bidder’s organ has been studied in several species and conditions (e.g., *Bufo ictericus* [164], *B. bufo* [161], *Bufo woodhousii* [165], and *Rhinella schneideri* [166]). This gonadal structure resembles ovarian tissue. In most species, no evident medulla and medullar cavity are observed (the medulla can be evident only during some developmental stages (e.g., *B. bufo* [161]). However, there are species with an identifiable cortex (with follicles at different stages of development) and medulla (with collagen fibers and blood vessels in some cases) (e.g., *B. ictericus* [164]). At pre-metamorphic stages, the oocytes in Bidder’s organ are similar to diplotenic ovarian oocytes, surrounded by a layer of follicular cells in both cases [167]. Therefore, it is not surprising that the expression profile of Bidder’s organ, although different, is more similar to developing ovaries than testes [163].

The pre-vitellogenic oocytes in Bidder’s organ do not mature, but undergo a degenerative process, ending with their reabsorption by the follicle cells [164,167,168]. The stock of stem germ cells (for successive annual oogenetic waves) is maintained as nests of germ cells at different stages of oogenesis. Low doses of estrogens can induce vitellogenesis in the bidderian oocytes, but not their maturation [168]. Treatments with different sex-steroids and antiandrogens reveal that the germ cells of Bidder’s organ have a strong commitment to the oogenetic pathway, probably related to their early entry into meiosis [169]. On the other hand, after removal of the testes by orchidectomy, Bidder’s organ becomes a functional ovary and the oocytes reach vitellogenic stages and complete maturation (e.g., *B. bufo* [168,170], *B. woodhousii* [165], and *Rhinella marina* [167]). Thus, the testes (androgens) inhibit the complete maturation of bidderian germ cells (probably inhibiting the response to gonadotropin), but not their proliferation and the oogenetic pathway [165].

The formation of Bidder’s organ may be related to localized high levels of retinoic acid in a region with early high expression levels of *Raldh2* (which catalyzes the synthesis of retinoic acid) and low levels of *Cyp26b1* (involved in the inactivation of retinoic acid) [171]. Increased levels of retinoic acid may be the reason for the early entry into meiosis and the advanced development observed in the bidderian germ cells, compared with those of the developing ovary [161].

The function of Bidder’s organ is still unknown. It has been considered a vestigial structure, or a morphological strategy to produce sexual cells for the reproduction of the species [164]. The most appealing hypothesis suggests that Bidder’s organ is a functional steroidogenic organ [163,172] that could be involved in the control of reproductive activity [162].

## 5. Genetic Control of Testicular Differentiation

Analysis of the genes expressed sex-specifically in the developing gonads of mammals [173], birds [174], reptiles [175,176], amphibians [177], and fish [178] reveals common genes between groups. However, differences in the expression patterns are also evident in different clades, with different genes showing heterochronic shifts between species (e.g., see Figure 4 in [179] for a comparison of the temporal onset of genes with sex-specific expression between turtles and mice during testis development) [177,179,180,181].

According to the current view, the differentiation of the gonads as testes or ovaries is not considered to be under the control of unidirectional pathways, but regulated by mutually exclusive (antagonistic) non-hierarchical networks [182]. These regulatory networks will activate one pathway while inhibiting the other to ensure that only one gonadal phenotype is achieved and maintained in adults. The molecular networks involved in testis differentiation in amphibians are not fully understood, as they have not been uncovered to the depth of those working in mice, chicken, reptiles, or fish (for a review, see [44,114,179]). The main reason may be related to the limitations of the amphibian species used as models to study the molecular networks involved in gonadal development. *X. laevis* can be sexed easily, but the interpretation of the gene expression results is difficult when differences between paralogous genes cannot be considered or have not been taken into account (e.g., comparing the *dmrt1* expression patterns in [119,183,184]). *X. tropicalis*, the diploid alternative to *X. laevis*, has three sex chromosomes, and the genetic sex of developing tadpoles cannot be easily established. *G. rugosa* is another widely studied species, although the existence of several populations with different sex chromosomes should be considered when the results are analyzed (e.g., differences in steroid sensitivity have been found between different populations [185]). Finally, regarding urodeles, *P. waltl* constitutes a well-known and widely used model. Its only limitation (and advantage from an evolutionary point of view) is that the results obtained may not be extrapolated to anurans.

Transcriptome analysis during gonadal differentiation has been performed in two anuran species (*X. laevis* and *X. tropicalis*) [177,186,187], whereas the information available on urodeles (the newt *Cynops orientalis*) is from adult gonads [181]. In addition, orthologs of genes known to be part of the gonadal differentiation pathway in mammals (*DMRT1*, *SOX9*, *FOXL2*, *AMH*, *DAX1*, *WNT4*, *SF1*, etc.) are also expressed during gonadal development in amphibians and have been analyzed in a wide number of anuran and urodelan species [119,135,171,183,188,189,190,191,192]. In general, these genes have expression profiles similar to those observed in other vertebrates, although differences between species regarding the onset and expression patterns reveal differences between the molecular networks, even in closely related species.

Gonadal expression patterns suggest that *dmrt1* has an important role in the differentiation of male gonads in amphibians (*X. laevis* [119,183,184,188,193], *G. rugosa* [194], *B. bombina*, *B. viridis*, *H. arborea*, *R. arvalis* and *R. temporaria* [188], *H. retardatus* [195]). The *dmrt1* gene was upregulated in differentiating amphibian testes [196,197,198,199], in the sex-reversed gonads of genetic females [194,200], and in female-to-male sex-reversed ZW gonads from *G. rugosa* tadpoles that were transgenic for *ar* and treated with low levels of testosterone [30]. On the other hand, in male-to-female sex reversal, *dmrt1* is downregulated. This has been described in the urodele *H. retardatus*, when genetic males were transformed into phenotypic females by means of high temperature treatments [195]. In *X. laevis*, the function of *dmrt1* in testis differentiation is antagonized by *dm-w*. The opposed roles of *dm-w* and *dmrt1* in testis differentiation are evident, as their overexpression induces ovarian and testicular development in ZZ and ZW gonads, respectively [73,74]. Furthermore, over-expression of the *dm-w* transgene in *X. laevis* ZZ gonads results in a high expression of *cyp19a1* and *foxl2* in developing gonads [74]. These two genes are involved in the differentiation of the ovary; both are upregulated in the gonads of female tadpoles or in the gonads of male-to female sex-reversed tadpoles [44,177,201]: *cyp19a1* (aromatase) converts testosterone into estrogen, leading to feminization, whereas *foxl2* can promote *cyp19a1* transcription in a *Xenopus* cell line [202].

*Dmrt1* is also required for spermatogonial stem cell maintenance in mice [203]. As in other vertebrates, in *X. laevis* two distinct promoters control the expression of *dmrt1.L* in Sertoli cells in males and in germ cells in both sexes [119]. The elimination of the transcripts derived from the germ cell-specific promoter causes a reduction in the number of germ cells in male and female gonads and results in female-to-male sex reversal in ZW tadpoles [119]. These results indicate that oocyte-produced signaling molecules could maintain the suppression of testis differentiation [204]. This has been demonstrated in zebrafish, as oocyte-produced *Bmp15* maintains *cyp19a1a* expression and estrogen production in granulosa cells, whereas *Bmp15*-deficient females became fertile males during the mid- to late-juvenile stage [205].

Steroids play important roles in sex differentiation in amphibians. Exogenous androgens or estrogens affect sexual differentiation during critical periods of development and cause the complete or partial sex reversal of the gonads [45]. Thus, it is not strange that one of the earliest changes in gene expression (taking place before any sign of gonadal differentiation) observed in developing tadpoles of *X. laevis* and *G. rugosa* concerns two steroidogenic enzymes, *cyp17a1* (a key enzyme involved in the production of male sex steroids) and *cyp19a1* (which controls the androgen/estrogen ratio by catalyzing the conversion of testosterone into estradiol) [196,197].

Other steroidogenic enzymes may be involved in gonadal differentiation. For example, the activity of 5α-reductase is higher in males than in females, leading to an increase in the conversion of testosterone into dihydrotestosterone (DHT). In this way, the ratio of DHT to estradiol could be responsible for male or female gonadal development [206].

The androgen receptor (*ar*) is involved in testis differentiation. In *G. rugosa,* the gene encoding *ar* is located on the sex chromosomes (Z, W, Y, and X) of this species [79]; and the W-linked *ar* copy has a lower level of expression than the Z-linked copy [80]. The Z-*ar* transgene, together with low doses of testosterone, can induce testis differentiation and the upregulation of *dmrt1* and *cyp17a1* in transgenic ZW tadpoles [30], suggesting that both genes could be a possible downstream target of Z-*ar* in this species.

*Sox9* is a key element in the testis pathway of mammals and birds, suggesting a conserved role for this gene in male differentiation [179,207]. However, *sox9* may not have a major role in testicular differentiation in amphibians. In *X. tropicalis*, *sox9* is detected in both male and female gonads after metamorphosis, indicating that this gene does not play a major role in early gonadal differentiation [189]. A similar situation was described in *G. rugosa* and *R. marina*, where *sox9* is expressed in both sexes during development, whereas upregulation in the testes was observed after metamorphosis [208,209]. In *X. laevis,* both *sox9* paralogs show higher expression in ZZ than ZW gonads during tadpole development [184], suggesting a possible role in testis differentiation. However, according to immunostaining results [184], it is also possible that these sequences are expressed in germ cells. In fact, expression of *sox9* in germ cells after metamorphosis has been described in *X. tropicalis* [189]. Thus, taking into account its participation in germ cell differentiation in fish [210], its role in somatic cell differentiation during testis development in birds and mammals must have been acquired later during evolution.

*Amh*, another key player in the testis-determining pathway in vertebrates, has a role in testis differentiation that is largely dependent on the clade analyzed. In mammals, *Sox9* cooperates with *Sf1* to activate *Amh* expression during testicular development [211]. In amphibians [184,209], as in chickens [212], *amh* precedes *sox9* expression. Upregulation of *amh* expression in the developing anuran testes has been described in *X. tropicalis*, *X. laevis,* and *G. rugosa*, among others [184,188,213,214]. In *X. tropicalis*, the Müllerian ducts begin to form between stages 57–66, according to [123], once the gonads have differentiated into testes or ovaries [213]. Thus, earlier expression in males could indicate a role in gonadal differentiation. In *P. waltl*, *amh* was also expressed at higher levels in the developing testes compared with the ovaries, with higher levels before and during gonadal differentiation in male gonads [191]. This profile of *amh* expression during the differentiation of amphibian testes could be related to its role in the control of germ cell proliferation, as described in fish [215,216,217]. In fact, in parabiosis experiments, in ZZ/ZW associations in *P. waltl*, the germ cell numbers in the ZW gonad were similar to those observed in the ZZ gonad and two-fold lower than in a control ZW gonad [218]. Similarly, mutations in the receptor of *amh* (*amhrII*) in the medaka caused the feminization of the gonads by altering proliferation at a specific stage of germ cell development [215,217]. This evidence suggests that *amh* had a primitive role in the proliferation and development of germ cells in early and adult gonads of both sexes, acquiring the function of Müllerian duct regression later during evolution [219].

Regarding the role of *amh* in Müllerian duct regression in amphibians, it is noteworthy that these ducts are maintained without differentiation in the male sex in urodeles [49,104] despite the expression and synthesis of *amh* in the developing testes [191]. Analysis of the expression of *amhr2* in the Müllerian ducts of the urodeles may explain its persistence, even though *amh* is expressed.

## 6. Conclusions

A general lack of knowledge exists about the sex-determining genes existing in amphibians. The available information on the sex chromosomes in this group predicts the existence of a wide variety of sex-determining genes. To improve our knowledge on amphibian testicular differentiation, it will be necessary to uncover more sex-determining genes in other species.

The undifferentiated amphibian gonads are organized into two domains: the cortex and the medulla. One of the first morphological signs of testis differentiation is the movement of germ cells from the cortex to the medulla (germ cells remain in the cortex in females). Among the main differences with testes in amniotes are: (1) the spermatogenesis takes place into the cyst; (2) all the germ cells that interact with one Sertoli cell in a cyst are at the same developmental stage; (3) there is a turnover of Sertoli cells in the adult due to the transient nature of the germinal epithelium. As consequence, the spermatogenic efficiency and the Sertoli supporting capacity of the testis are higher compared to amniotes.

Amphibians do not resemble fish in regard to their extreme testis plasticity, although examples of testis plasticity can be found in urodelans, in which new testis lobes develop in adult life. Furthermore, ovarian-like structures with pre-vitellogenic oocytes (Bidder’s organ) persist attached to the testes in bufonid males. The testis has an inhibitory effect on the differentiation of Bidder’s organ as an ovary, but no lethal effect on the germ cells.

The gene networks controlling testis differentiation in amphibians are closer to those in fish than those in birds and mammals. *dmrt1*, *cyp19a1*, *foxl2*, and *cyp17a1* are major players in controlling the gonadal development of amphibians. Establishing relations between these and other elements will require functional analysis. Amphibians are emerging as interesting models to discover how mutually exclusive networks evolved in vertebrates to control gonadal differentiation and how new sex-determining genes are frequently added to these networks in different species.

## Data Availability

Data sharing not applicable.
